# Extensive evaluation and classification of low‐cost dust sensors in laboratory using a newly developed test method

**DOI:** 10.1111/ina.12615

**Published:** 2019-11-12

**Authors:** Kang‐Ho Ahn, Handol Lee, Hae Dong Lee, Sang Chul Kim

**Affiliations:** ^1^ Department of Mechanical Engineering Hanyang University ERICA Ansan Korea; ^2^ Korea Conformity Laboratories Geumcheon-Gu, Seoul Korea

**Keywords:** embedded type sensor, exponentially decaying particle concentration, lighting source, low‐cost dust sensor, module type sensor, standalone type sensor

## Abstract

An extensive evaluation of low‐cost dust sensors was performed using an exponentially decaying particle concentration. A total of 264 sensors including 27 sensors with light‐emitting diodes (LEDs) and 237 sensors with laser lighting sources were tested. Those tested sensors were classified into 4 groups based on the deviation from the reference data obtained by a reference instrument. The response linearities of all the tested samples for PM_1_, PM_2.5_, and PM_10_ were in excellent agreement with the reference instrument, except a few samples. For the measurements of PM_1_ and PM_2.5_, the lighting source, that is, LED or laser, did not show any significant difference in overall sensor performance. However, LED‐based sensors did not perform well for PM_10_ measurements. The 32, 24, and 16% of all the tested sensors for PM_1_, PM_2.5_, and PM_10_ measurement, respectively, are in the category of Class 1 (reference instrument reading ± 20%) requirement. The performance of the low‐cost dust sensors for PM_10_ measurement was relatively less satisfactory.

**Practical Implications**

- There are lots of low‐cost dust sensors available in the market or used by many researchers and citizen scientists for air quality evaluations; however, the reliability of these sensors has been questioned due to the absence of a standardized calibration method.
- This study suggests the newly developed test method for the evaluation of low‐cost dust sensors, and it classifies the 264 sensors into 4 groups based on the accuracy compared with the reference instrument.
- The developed test method can be used for the evaluation of the response time of the low‐cost dust sensors tested under the fast‐changing concentration conditions, and through the introduced test method, one can select the proper sensors for their specific purpose and calibrate the sensors quickly.

## 1 INTRODUCTION

Air quality that is considerably related to climate change, human health, and other environmental conditions can be assessed by measuring particulate matter (PM) concentrations. Air pollution associated with PM concentrations has been studied significantly on its adverse effects.^1^, ^2^, ^3^, ^4^ Fine airborne particles, PM_2.5_ (particle size less than or equal to 2.5 μm), have been considered one of the highest health risks, causing numerous diseases including lung cancer, arrhythmia, asthma, pneumonitis, and cardiovascular mortality.^5^, ^6^, ^7^, ^8^, ^9^, ^10^, ^11^ The first key factor for effective management of air pollution is to continuously monitor air quality by measuring PM concentrations. Most of the government agencies in the world employ sparsely distribute monitoring stations equipped with expensive and high‐quality monitoring systems. However, the stations cannot effectively and accurately represent the pollutant gradients within cities, and it is even more difficult to assess air quality in indoor environment.^12^, ^13^, ^14^ Due to this limitation in the coverage of the monitoring stations, recently many industries and researchers have been developing real‐time monitoring systems for large networks.

Recently, many low‐cost sensors based on light scattering techniques with optical systems have been widely developed and employed to evaluate air quality with much higher spatial resolution.^15^ Moreover, the new sensors have been adopted by many researchers and citizen scientists in many applications to collect personal exposure and mobile monitoring data in hot spots.^16^, ^17^, ^18^, ^19^ However, several issues such as little information from manufacturers, simplified measurement, and noise handling techniques are growing concerns on reliability and accuracy of the low‐cost sensors.^20^ Besides, relatively little investigation on the performance of these low‐cost sensors have been conducted, and there is still lack of standard calibration and evaluation methods for the sensors.

Several researchers adopted their own calibration and evaluation procedures to test the low‐cost sensors including co‐location measurements in ambient environment and lab‐scale measurements using standard test particles or ambient airborne particles.^15^, ^18^, ^20^, ^21^, ^22^, ^23^, ^24^, ^25^ However, the accuracy of measurement data has always been questioned by many researchers and scientists,^15^, ^26^, ^27^ and the limited number of sensors have been assessed due to the time‐consuming evaluation process, which might not be able to represent the overall performance of hundreds of low‐cost sensors in the commercial market. Besides, the lack of standardized methods for performance evaluation of low‐cost sensors results in difficulties in intercomparisons of the sensors evaluated in different studies.^28^ Therefore, a standard test procedure should be required for these overflowing low‐cost sensors.

In the year 2015, there was a big dispute in Korea about the accuracy of low‐cost dust sensors. Since then many scientists and industrial people have been collaborating to establish a standardized method in testing a dust sensor. As a result, the Korean industrial standard “SPS‐C KACA 0027‐7269:2018” for a low‐cost dust sensor test was introduced in May 30, 2018. Based on this standardized procedure, we tested the low‐cost dust sensor extensively from June to December 2018, and we classified the sensors into 4 groups based on the accuracy of the sensor measurements by comparing a reference instrument. In this study, 264 sensors with three different types according to their deployment conditions were tested, that is, a “module type” sensor that is half‐finished and cannot be used as it is a “standalone type” sensor that is ready to use and an “embedded type” sensor that is installed inside an air purifier or an air conditioner. Based on this classification, 53 module type, 126 standalone type, and 85 embedded type sensors, that is, total 264 sensors from 23 manufactures were systematically evaluated. Since the low‐cost dust sensors are operated by detecting a scattered light coming from a particle, we checked the sensor's lighting source effect on its performance. The 27 and 237 sensors out of the total 264 sensors have a light‐emitting diode (LED) and a laser as their lighting source, respectively. It should be noted that this paper systematically addressed the performance of hundreds of sensors according to their PM concentration measurements at a glance. Our findings from the results based on the standardized sensor evaluation are expected to provide important insights and valuable guidelines to effectively select and develop low‐cost sensors.

## 2 TEST METHOD

**Figure 1:**
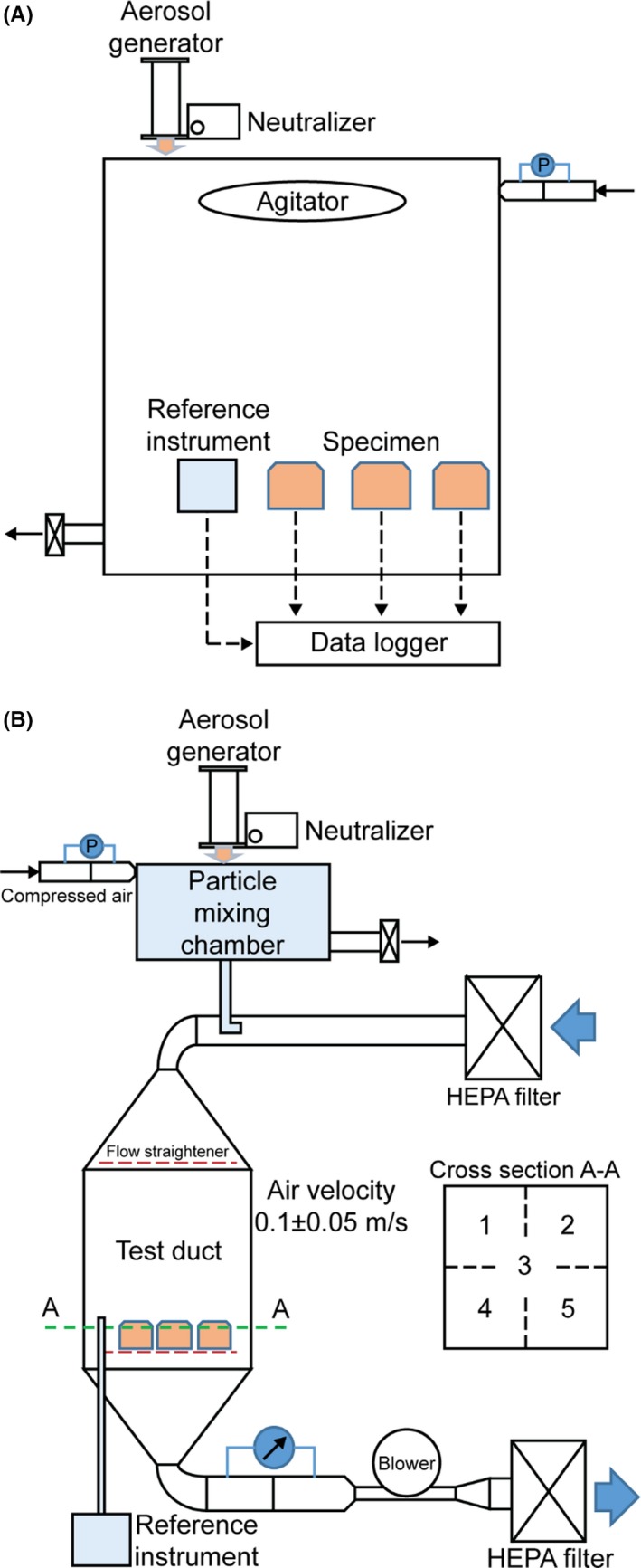
Schematics of low‐cost dust sensor evaluation systems: (A) chamber test; (B) low‐speed duct test

Basically, we adopted an exponentially decaying particle concentration for the evaluation of low‐cost dust sensors. The advantage of this concentration condition is that the testing time is relatively short, mostly taking less than 10 minutes, compared with other test methods using a constant particle concentration, which usually takes more than an hour.^29^ Therefore, in this study, two types of test systems using the exponentially decaying particle concentration were applied. The first one is a chamber system as shown in Figure 1A. This method is very useful when testing an air purifier with an embedded type of a low‐cost dust sensor. Due to the purifying process of the air purifier removing particles inside the test chamber, the particle concentration in the chamber will decrease exponentially as a function of time. The chamber dimension in Figure 1A is 5 × 5 × 2 m^3^. For testing a dust sensor in an air purifier using the chamber system shown in Figure 1A, the introduced particles in the chamber are well mixed by the air coming from the air purifier and if necessary, an auxiliary agitating fan can be used to ensure the uniform particle concentration in the chamber. In this case, one must be careful about the airflow velocity near the dust sensor. If the flow speed is high near the sensor then the dust sensor may not function properly.^30^ The second test system is to employ a low air‐speed duct with an exponentially decaying particle concentration as shown in Figure 1B. This system performs well for the test of a low‐cost dust sensor with a small size and high sensitivity to an airflow around it. The details in the low‐speed duct test method are well described in Kang et al^30^ Briefly, generated particles by an atomizer are introduced to a particle mixing chamber until the particle concentration in the chamber reaches a certain level, and then the path between the aerosol generator and the mixing chamber is disconnected. After this step, only the clean air is supplied to the particle mixing chamber, and the particles with an exponentially decaying concentration are introduced to the test duct system. We used a flow straightener with a typical honeycomb structure before the test section to obtain the flow uniformity. The uniformity of flow velocity and particle concentration in the test section was confirmed before each test.

**Figure 2:**
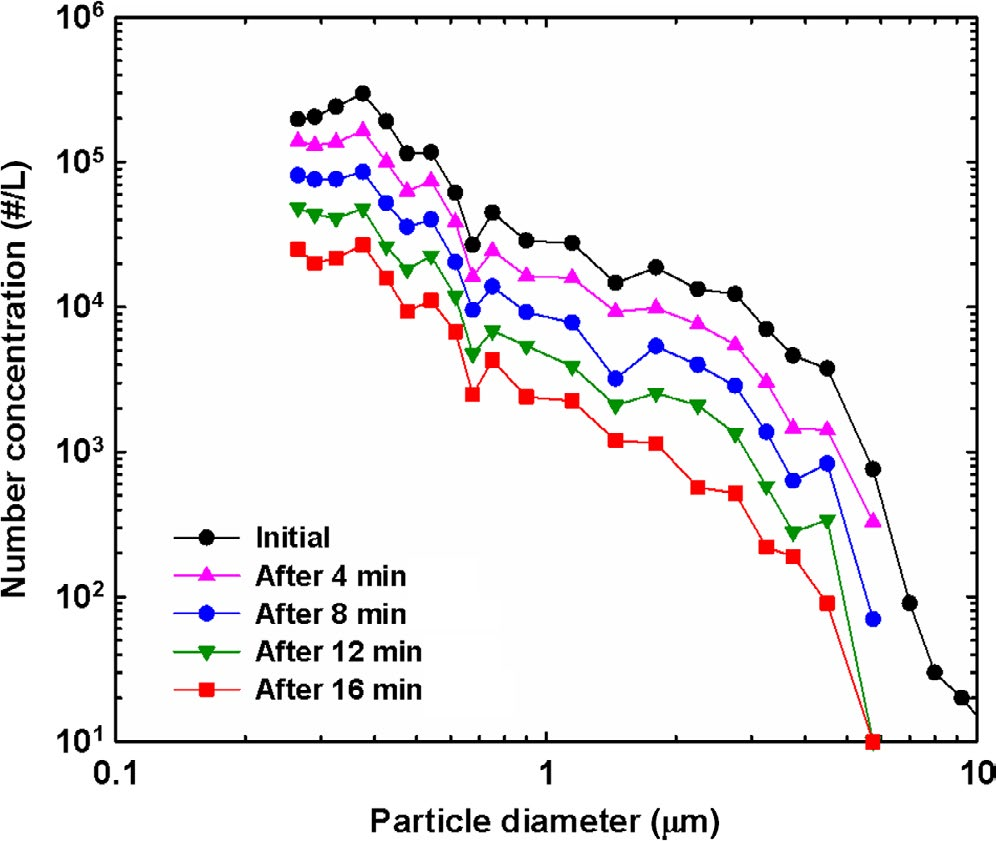
Potassium chloride particle size distributions as a function of elapsed time with an exponentially decaying particle concentration. The time interval is 4 min

For test particles, 5 wt% potassium chloride (KCl) solution was used to generate KCl particles by an atomizer for both test systems. The exemplary size distributions of the generated KCl particles as time elapses with 4 minutes of the time interval are shown in Figure 2. The size distributions were measured by a Grimm 1.209 dust monitor (Model 1.209, Grimm Aerosol Technik Company), and this was used as a reference instrument in this study. The number mean diameter was 0.44 ± 0.02 μm, and the geometrical standard deviation of 1.62 ± 0.05 was maintained during the entire testing periods for 6 months, that is, from June to December, 2018. Tested low‐cost sensors measured particle concentrations, and the data were compared to the data simultaneously obtained by the reference instrument.

**Figure 3:**
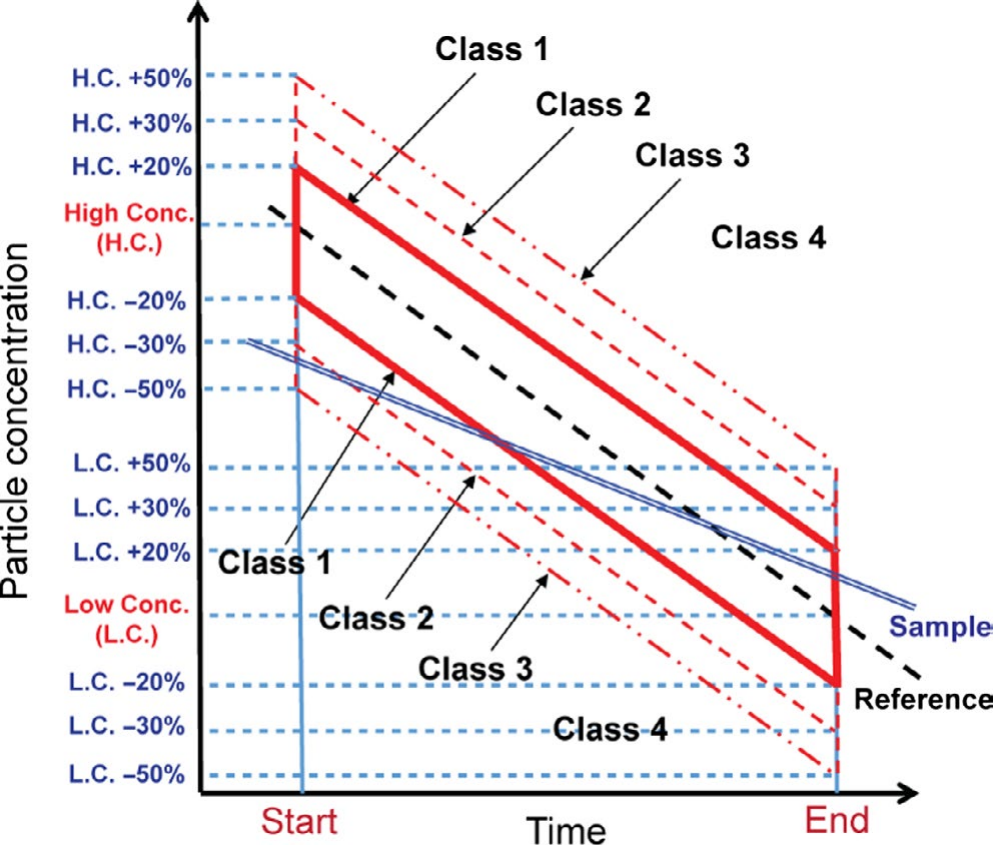
Classification and acceptance limits of low‐cost sensor performance

The sensor performance was classified into 4 groups based on the deviation from the reference data as shown in Figure 3. For the evaluation of the sensors, we focused on their measurement accuracy and response characteristic. The accuracy of the sensors was estimated by the absolute concentration data at the lowest and highest concentrations, and the response characteristics to the rapidly changing concentration were assessed by the ratio of the slope obtained by the sensors to the one from the reference instrument.

The Class 1 acceptance limit is represented by a red solid line parallelogram in a semi‐log graph. This is equivalent to the inside of ±20% of the regression line of the reference data (black dashed line). It should be noted that due to the exponentially decaying particle concentration, the concentration is represented in log scale on the y‐axis. In the same manner, we set the limits as ±30% and ±50% of the reference data for Class 2 and Class 3, respectively. Class 4 is the one beyond the Class 3 acceptance criteria. To clarify the classification method, we showed an example in Figure 3. It can be seen that the measurement data of the test sensor for the highest and lowest concentrations during the test period meet the criteria for Class 3 and Class 1, respectively. In this case, we classify this low‐cost dust sensor as Class 3. It should be mentioned that if there are experimental errors in the particle generation or flow system, the regression data obtained by the reference instrument shown in Figure 3 will not be straight in a semi‐log graph.

## 3 RESULTS AND DISCUSSION

**Figure 4:**
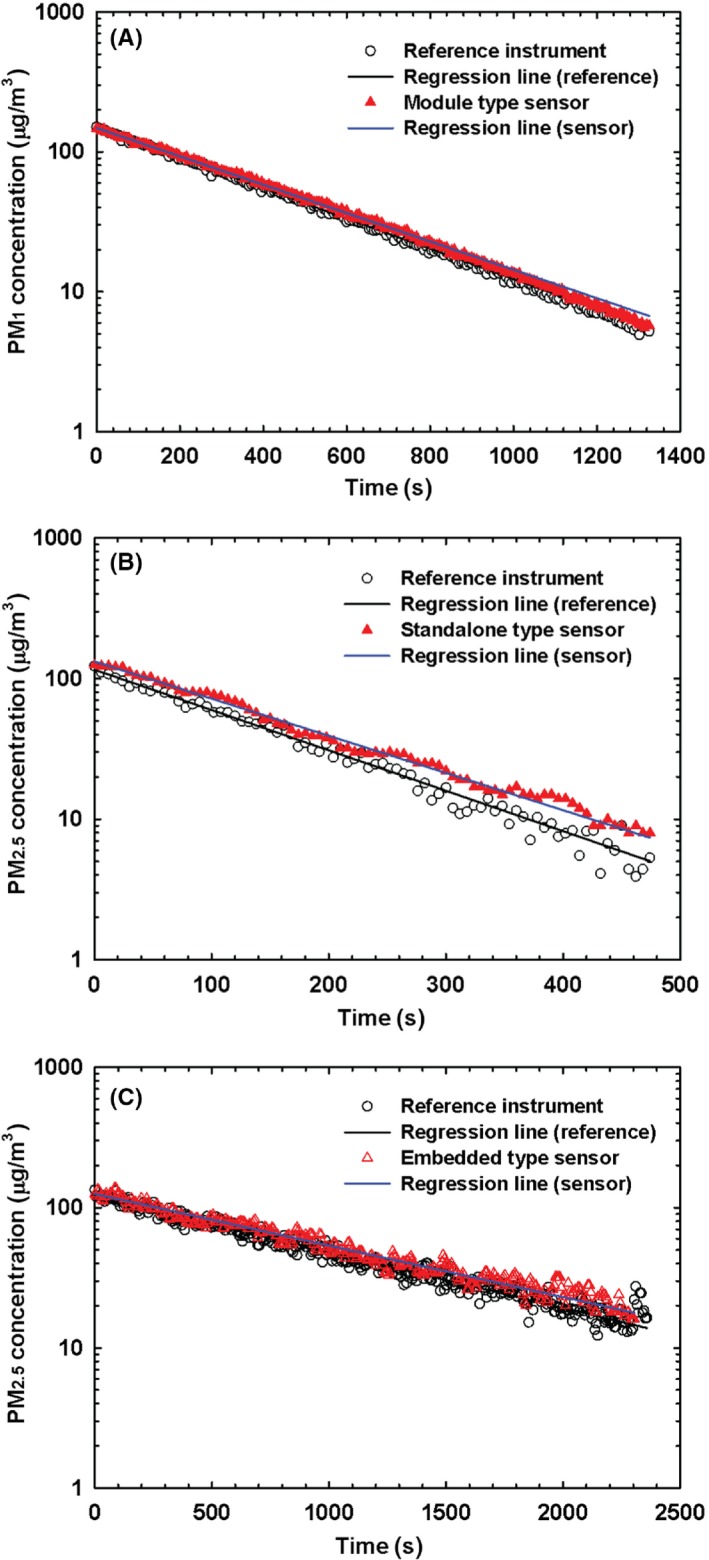
Exemplary measurement data obtained by the (A) module (PM_1_), (B) standalone (PM_2.5_), and (C) embedded (PM_2.5_) type sensor

Some of the sample test results are shown in Figure 4. Figure 4A‐C are the sample test results for a “module type” sensor, “standalone type” sensor, and “embedded type” sensor, respectively. Based on the criteria introduced in the previous section, the tested sensors in Figure 4A‐C were classified to Class 1, Class 3, and Class 2, respectively.

**Figure 5:**
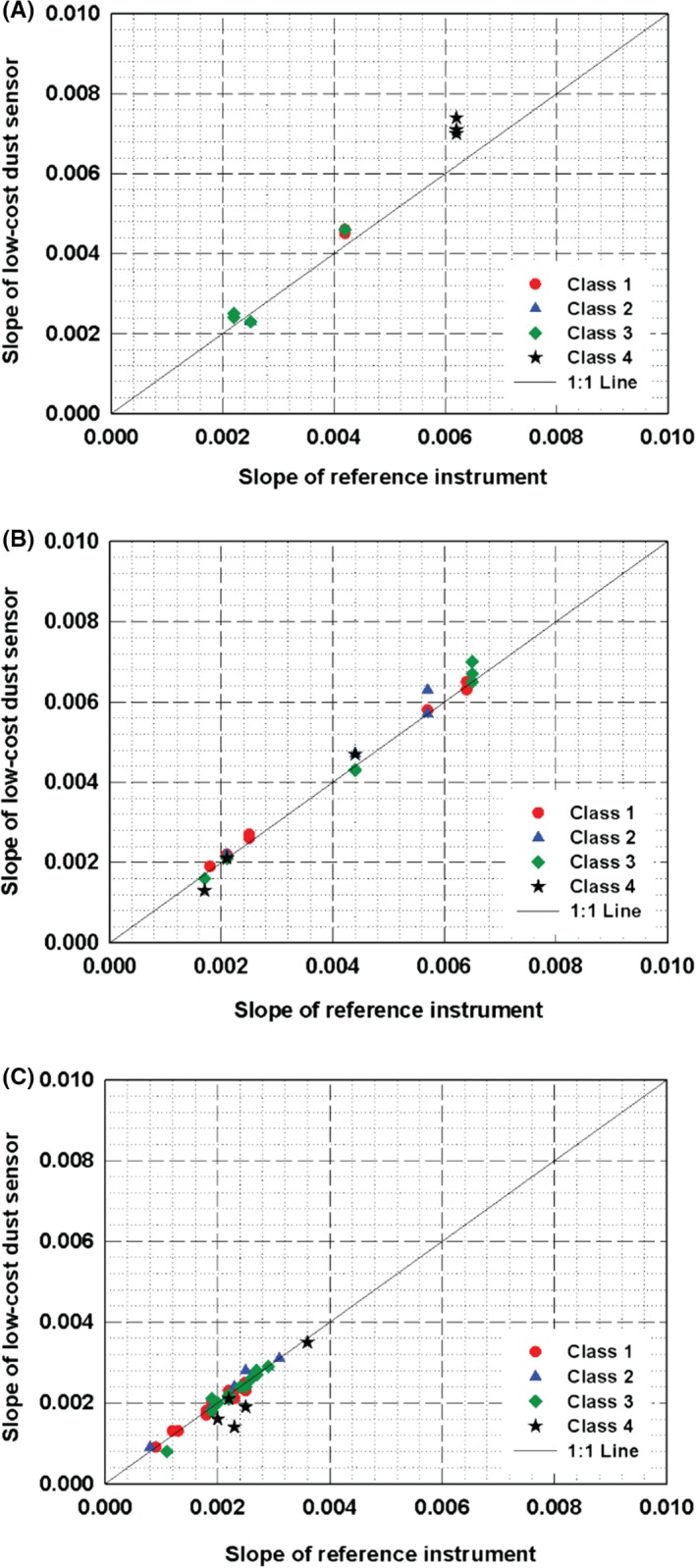
Slopes of the regression lines for PM_1_ measurement obtained by test sensors (78 samples) and reference instrument: (A) module; (B) standalone; (C) embedded type sensor

Figure 5 represents the slopes obtained from the measurements using 78 PM_1_ sensors including all three types, that is, module, standalone, and embedded types. It is clearly seen that the absolute values of the slopes obtained from the test sensors classified as Class 1, 2, and 3 are in good agreement with those from the reference instrument regardless of sensor types, lying on the 1:1 line. However, the obtained slopes from some of Class 4 sensors are slightly deviated from the reference data, for example, black stars in Figure 5A.

**Figure 6:**
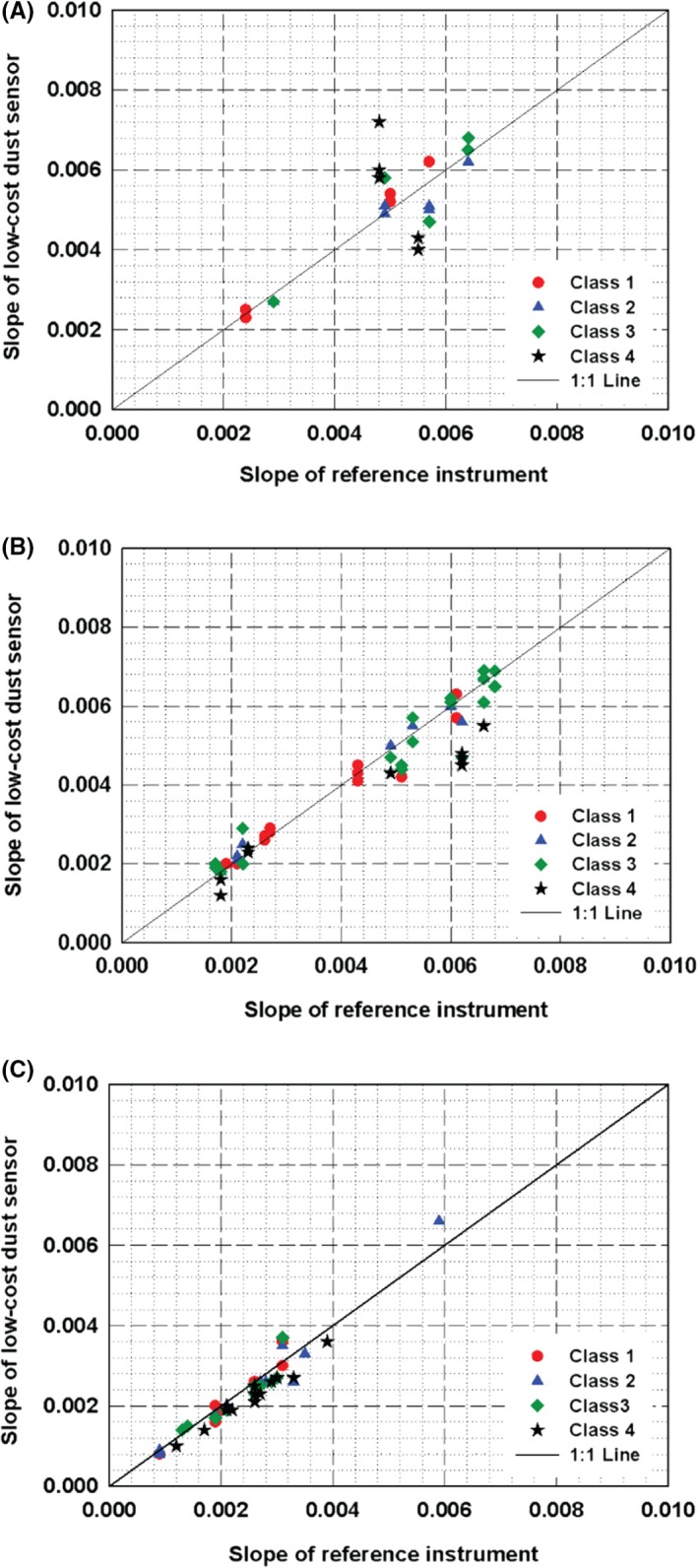
Slopes of the regression lines for PM_2.5_ measurement obtained by test sensors (125 samples) and reference instrument: (A) module; (B) standalone; (C) embedded type sensor

The linearity comparison for 125 PM_2.5_ sensors with the three types was also performed, and the results are shown in Figure 6. Except for some of Class 4 sensors, the other low‐cost dust sensors have the similar slopes to the reference instrument, indicating the response characteristics of the sensors to the exponentially changing concentration are generally good. The degree of deviations from the 1:1 line shown in the module type sensors, that is, Figure 6A, was found to be larger than the other two types of sensors, especially when compared to the embedded type sensors in Figure 6C.

**Figure 7:**
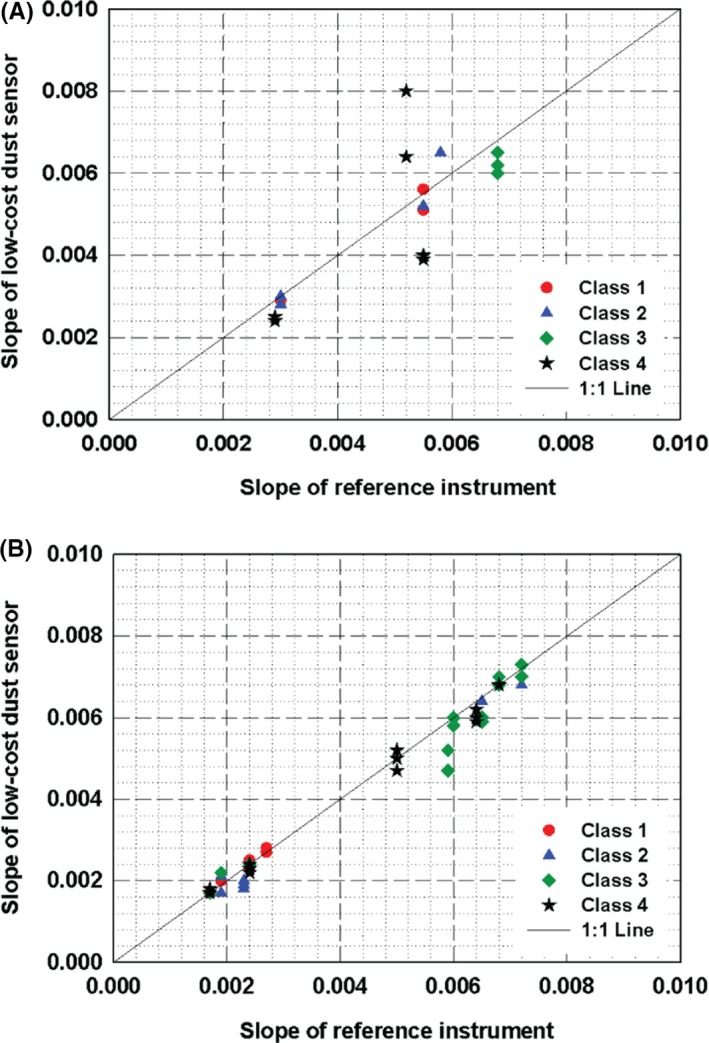
Slopes of the regression lines for PM_10_ measurement obtained by test sensors (61 samples) and reference instrument: (A) module; (B) standalone type sensor

The results for PM_10_ measurements are represented in Figure 7, but due to some difficulties in manufacturing process, which might be related to failure of the satisfaction of certain criteria, embedded type sensors were not available during the test period. Therefore, Figure 7 only shows the results for the module and standalone types of sensors. We found that most of the Class 4 sensors with the module type showed significant deviations in slope while relatively good linearity was seen for the standalone type sensors.

**Figure 8:**
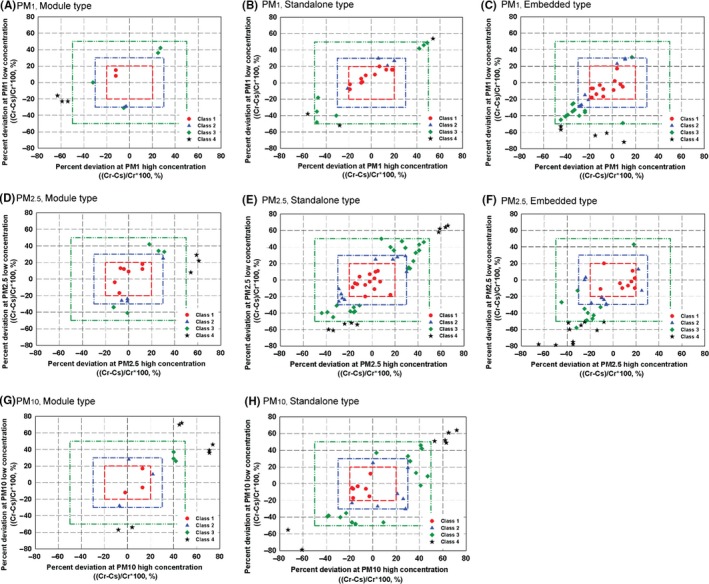
Deviations of particulate matter (PM) concentration measurements from the reference data at high and low particle concentrations. *C_s_* and *C_r_* are the concentration measured by a test sensor and reference instrument, respectively: (A) PM_1_, module; (B) PM_1_, standalone; (C) PM_1_, embedded; (D) PM_2.5_, module; (E) PM_2.5_, standalone; (F) PM_2.5_, embedded; (G) PM_10_, module; (H) PM_10_, standalone type sensor

In addition to the evaluation of linearity, we can clearly evaluate the degree of deviation for the measurements of high and low particle concentrations from the reference data. Figure 8 quantitatively represents the degree of deviation of PM_1_ (Figure 8A‐C), PM_2.5_ (Figure 8D‐F), and PM_10_ (Figure 8G,h) measurements relative to the reference instrument at the high and low concentrations. If a data point is placed within ±20% of the reference data at both high and low concentration, the sensor is classified as Class 1, which is depicted as a circular solid dot in a red rectangle. In the same way, the limit of Class 2 and 3 is represented as a blue and dark red rectangle, respectively. To be noted, some of the sensor data points that are out of the most outer boundaries, that is, over ±80%, are not shown. From the way of representing the data as shown in Figure 8 based on the testing method introduced in this study, one can easily evaluate the performances of sensors according to particle size and concentration.

The performance classifications of test sensors are summarized in Table 1 for different sensor types, that is, module, standalone, and embedded types. According to the results shown in Table 1, about one quarter of the low‐cost dust sensors regardless of sensor type were classified into Class 1. As seen in the table, a considerable portion of the module type sensors, that is, 32.1%, belongs to Class 4, indicating the less accuracy compared with the standalone and embedded type sensors. Therefore, it can be concluded that standalone and embedded type sensors were calibrated better by the final manufactures than the half‐finished module manufactures.

**Table 1:** The ratio of low‐cost dust sensors for each Class based on particulate matter: (a) module; (b) standalone; (c) embedded type sensor

	Class 1	Class 2	Class 3	Class 4	Total
(a) Module type					
PM_1_	2 (3.8%)	2 (3.8%)	4 (7.5%)	3 (5.7%)	11 (20.8%)
PM_2.5_	8 (15.1%)	4 (7.5%)	6 (11.3%)	6 (11.3%)	24 (45.3%)
PM_10_	3 (5.7%)	4 (7.5%)	3 (5.7%)	8 (15.1%)	18 (34.0%)
Total	13 (24.5%)	10 (18.9%)	13 (24.5%)	17 (32.1%)	53 (100%)
(b) Standalone type					
PM_1_	10 (7.9%)	4 (3.2%)	8 (6.3%)	3 (2.4%)	25 (19.8%)
PM_2.5_	15 (11.9%)	10 (7.9%)	22 (17.5%)	11 (8.7%)	58 (46.0%)
PM_10_	7 (5.6%)	7 (5.6%)	15 (11.9%)	14 (11.1%)	43 (34.1%)
Total	32 (25.4%)	21 (16.7%)	45 (35.7%)	28 (22.2%)	126 (100%)
(c) Embedded type					
PM_1_	13 (15.3%)	9 (10.6%)	15 (17.6%)	5 (5.9%)	42 (49.4%)
PM_2.5_	9 (10.6%)	10 (11.8%)	11 (12.9%)	13 (15.3%)	43 (50.6%)
Total	22 (25.9%)	19 (22.4%)	26 (30.6%)	18 (21.2%)	85 (100%)

**Figure 9:**
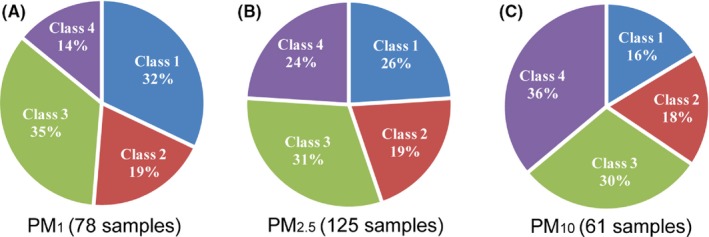
Classification of low‐cost sensors based on particulate matter (PM): (A) PM_1_ (78 samples); (B) PM_2.5_ (125 samples); (C) PM_10_ (61 samples)

Figure 9 represents the percentages of sensor grade depending on PM for all test sensors. The accuracy of the low‐cost dust sensors is higher for detecting smaller sized particles, that is, PM_1_, compared with PM_2.5_ and PM_10_. For instance, the Class 1 satisfaction ratio for PM_1_, PM_2.5_, and PM_10_ is 32%, 26%, and 16%, respectively. From the result, low‐cost dust sensors cannot detect larger particles efficiently, which should be a main part needed to be improved near future. It should be noted that theoretically the optic system can easily detect large particles due to higher scattering intensity. However, these test results showed opposite trend, and one possible explanation is that the low particle sampling efficiency and the significant transportation losses for larger particles inside a sensor. Therefore, the findings in this study also enlighten the importance of the proper designs of the sampling port and flow channel for minimizing the particle losses, which might affect the sensor performance significantly.

**Figure 10:**
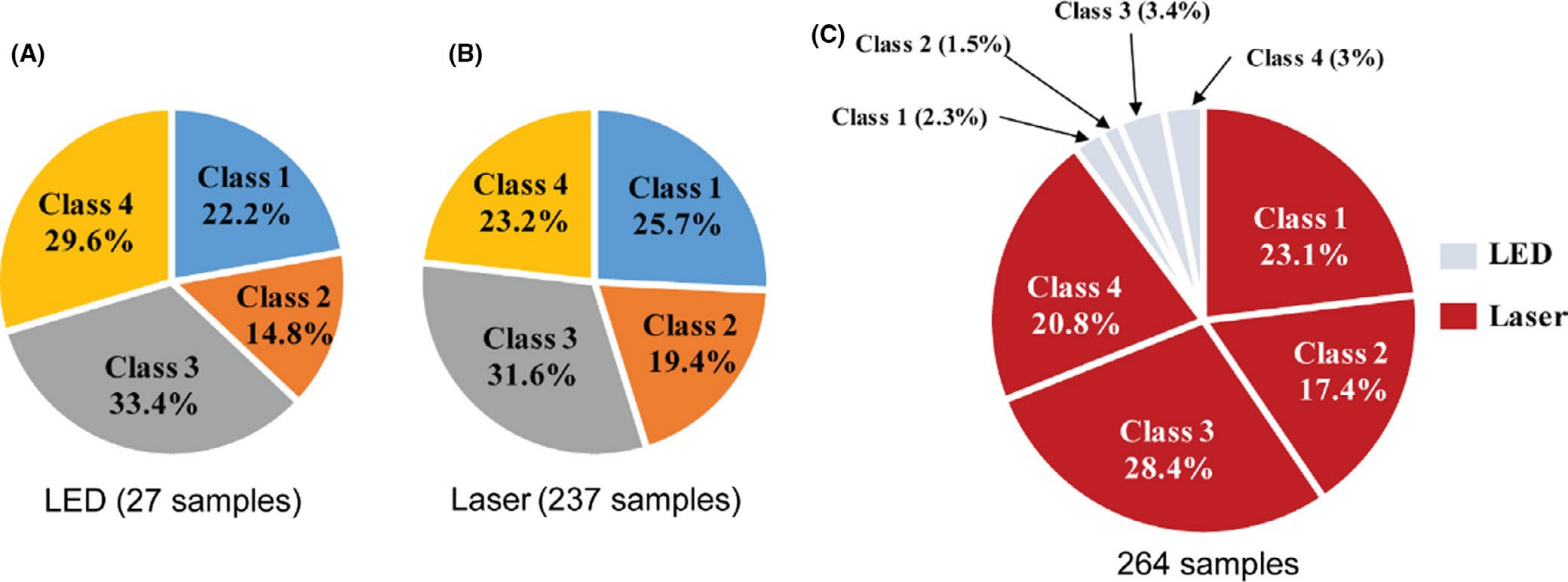
Classification of low‐cost sensors based on light source: (A) LED (27 samples); (B) laser (237 samples); (C) total 264 samples

We classified the sensors’ performance according to the type of light source in the sensors, that is, LED or laser, shown in Figure 10. Only 27 samples adopted an LED as an illumination source, and most of the sensors used a laser, that is, 237 samples. As it is shown in Figure 10, no significant difference on sensor performance was found.

**Figure 11:**
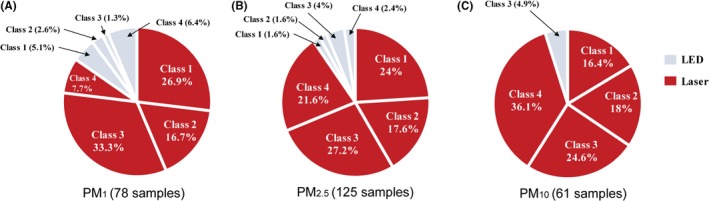
Classification of low‐cost sensors based on particulate matter (PM) and light source: (A) PM_1_ (78 samples); (B) PM_2.5_ (125 samples); (C) PM_10_ (61 samples)

We also estimated the ratios of the low‐cost sensors according to PMs in Figure 11, and detailed values are summarized in Table 2. Interestingly, the effect of a light source on detecting different sized PMs was observed from the results showing that the low‐cost sensors with an LED as a light source can hardly function on PM_10_ measurement as shown in Figure 11C. This may be caused by the sampling mechanism of the LED type sensors. LED type sensors usually employ heating registers as an air mover. This register type air mover usually generates very low air speed inside the sensor that can hardly transport large‐sized particles into the sensing zone.

**Table 2:** The number and ratio of low‐cost dust sensors for each Class based on light source: (a) PM_1_; (b) PM_2.5_; (c) PM_10_

	Class 1	Class 2	Class 3	Class 4	Total
(a) PM_1_					
LED	4 (5.1%)	2 (2.6%)	1 (1.3%)	5 (6.4%)	12 (15.4%)
Laser	21 (26.9%)	13 (16.7%)	26 (33.3%)	6 (7.7%)	66 (84.6%)
Total	25 (32.1%)	15 (19.2%)	27 (34.6%)	11 (14.1%)	78 (100%)
(b) PM_2.5_					
LED	2 (1.6%)	2 (1.6%)	5 (4%)	3 (2.4%)	12 (9.6%)
Laser	30 (24.0%)	22 (17.6%)	34 (27.2%)	27 (21.6%)	113 (90.4%)
Total	32 (25.6%)	24 (19.2%)	39 (31.2%)	30 (24%)	125 (100%)
(c) PM_10_					
LED	0	0	3 (4.9%)	0	3 (4.9%)
Laser	10 (16.4%)	11 (18.0%)	15 (24.6%)	22 (36.1%)	58 (95.1%)
Total	10 (16.4%)	11 (18%)	18 (29.5%)	22 (36.1%)	61 (100%)

In general, the sensor performance of the LED‐based module type sensors is much less satisfactory than the embedded type sensors. This may imply that the sensors embedded in air purifiers have better algorithms or calibrations than the half‐finished module sensors. The performance of the low‐cost dust sensors according to the light source and the sensor types is summarized in Table 3. The assessment procedure for different types of sensors and the data shown in this study will provide manufacturers and users with valuable insights on the performance of low‐cost dust sensors.

**Table 3:** The number of low‐cost dust sensors for each Class based on sensor type and light source: (a) LED; (b) laser

	Class 1	Class 2	Class 3	Class 4	Total
(a) Module type					
PM_1_	0	0	0	3	3
PM_2.5_	0	1	2	0	3
PM_10_	0	0	3	0	3
Embedded type					
PM_1_	4	2	1	2	9
PM_2.5_	2	1	3	3	9
PM_10_	0	0	0	0	0
(b) Module type					
PM_1_	2	2	4	0	8
PM_2.5_	6	6	3	6	21
PM_10_	3	4	0	8	15
Standalone type					
PM_1_	10	4	8	3	25
PM_2.5_	15	9	23	11	58
PM_10_	7	7	15	14	43
Embedded type					
PM_1_	9	7	14	3	33
PM_2.5_	7	9	8	10	34
PM_10_	0	0	0	0	0

## 4 CONCLUSION

Low‐cost dust sensor performance has been tested using an exponentially decreasing particle concentration that is recommended in Korean industrial standard “SPS‐C KACA 0027‐7269:2018.” About 264 sensor samples were tested and classified into 4 different groups, Class 1, 2, 3, and 4, depending on their performance, which is evaluated by comparing with an optical particle counter used as a reference instrument. Most of the sensors showed a very good linearity (slope of concentration measurement data) with the reference data. About one quarter of the tested sensors satisfied the Class 1 acceptance limit. However, the accurate PM_10_ measurement by using the low‐cost sensors was found to be relatively difficult to achieve, which might be caused from the difficulty in transporting large particles to detection zones. The comprehensive assessment presented in this study should be widely adopted for the further improvement of low‐cost dust sensors in this high demand.
